# Drift current-induced tunable near-field energy transfer between twist magnetic Weyl semimetals and graphene

**DOI:** 10.1515/nanoph-2023-0345

**Published:** 2023-10-05

**Authors:** Qijun Ma, Xue Chen, Qisen Xiong, Leyong Jiang, Yuanjiang Xiang

**Affiliations:** School of Physics and Electronics, Hunan University, Changsha 410081, China; School of Physics and Electronics, Hunan Normal University, Changsha 410081, China

**Keywords:** drift current, magnetic Weyl semimetals, graphene, nonreciprocal photon occupation number, nonreciprocal surface mode, near-field radiative heat transfer

## Abstract

Both the nonreciprocal surface modes in Weyl semimetal (WSM) with a large anomalous Hall effect and the nonreciprocal photon occupation number on a graphene surface induced by the drift current provide a promising way to manipulate the nonreciprocal near-field energy transfer. Interestingly, the interactions between nonreciprocities are highly important for research in (thermal) photonics but remain challenging. In this study, we theoretically investigated the near-field radiative heat flux transfer between a graphene heterostructure supported by a magnetic WSM and a twist-Weyl semimetal (T-WSM). The nonreciprocal surface mode could be changed by the separation space between two Weyl nodes and the twist angle. Notably, we found that in the absence of a temperature difference between two parallel plates, nonequilibrium fluctuations caused by drift currents led to the transfer of near-field radiative heat flux. Furthermore, these nonreciprocal surface modes interacted with the nonreciprocal photon occupation number in graphene to achieve flexible manipulation of the near-field heat flux size and direction. Additionally, graphene adjustable flux in the case of a temperature difference between the two plates was also discussed. Our scheme can provide a reference for near-field heat flux regulation in nonequilibrium systems.

## 1 Introduction

When the distance between two objects is less than the characteristic wavelength of thermal radiation specified by Wien’s displacement law, near-field radiative heat transfer (NFRHF) occurs as the radiative heat flux exceeds the blackbody limit by several orders of magnitude [1, 2]. In the near-field range, evanescent coupling can make it easier for photons to pass through a vacuum gap. Moreover, the excitation of some surface electromagnetic modes can also further enhance the near-field heat radiation. Over the past few decades, near-field heat radiation has proven to be very popular in several fields due to its advantages over blackbody radiation, including thermal imaging [3], highly efficient thermal management [4], thermal rectification [5], and photon transformation [6]. The potential applications of near-field heat radiation in the abovementioned and other fields have attracted much attention from researchers. In addition to applying magnetic fields in magneto-optical materials [7] and changing surface polarons based on the dielectric function of materials [8–10], other options for the effective control of near-field heat flux are available. For example, the regulation of heat flux transfer in a three-body system is achieved through relative rotation of the modulator [11–14].

In recent years, graphene, a two-dimensional (2D) layered material, has also received much attention and provided to significant results in the field of near-field heat radiation due to its characteristics, such as adjustable Fermi energy level [15], convenient dynamically adjustable optical conductivity [16], and ultrawide working bandwidth [17]. Moreover, the graphene-supported frequency tunability of plasma [18] and hyperbolic graphene plasmons [19] also play a positive role in enhancing and regulating near-field thermal radiation. In particular, graphene, relying on ultrahigh electron mobility, can regulate the heat flux by applying a current with a certain drift velocity to its surface. Peng et al. demonstrated the presence of a current-induced near-field heat flux in double-layer graphene from the perspectives of fluctuational electrodynamics (FE) and nonequilibrium Green’s function (NEGF) [20]. Zhang et al. established an asymmetric photonic transmission model by applying an adjustable drift current across the graphene sheet [21]. Through a drift current on the graphene surface and an in-plane magnetic field in the magneto-optic medium, Tang et al. achieved control of the near-field energy transfer direction and produced a uneable thermoelectric current in graphene. All these studies confirm that graphene added with drift current has a large tuning potential; thus, the relevant near-field heat radiation scheme is one of the feasible ways to achieve the corresponding thermal radiation devices. However, the regulation scheme of the near-field heat radiation at the micro/nano level is difficult to achieve.

According to thermodynamic laws, the equivalence of emissivity and absorptivity mentioned in Kirchhoff’s law of radiation is not needed in a thermal radiation system, because this is only a requirement for the Lorentz reciprocity. Therefore, in thermodynamics, we can break the symmetry of the dielectric tensor to remove the restraints imposed by Kirchhoff’s law and this provides a new concept for one-way transmission. At present, some schemes based on the above concepts are available to achieve control of near-field radiative heat flux transport, such as the negative thermal magnetoresistance effect [22], thermal router [23], and photon thermal Hall effect [24]. Targeting the difficulties in reciprocal systems, the Weyl semimetal (WSM), a new favorite in this area, has its own advantages in terms of material properties in breaking reciprocity. Due to the WSM’s unique topological nontrivial electronic state and inherent time-reversal symmetry breaking, the Weyl node distance in momentum space acts as an effective applied magnetic field, thus leading to a large anomalous Hall effect [25–27]. Therefore, the WSM can break Lorentz reciprocity and shows particular advantages in controlling thermal radiation; these has been widely reported in axion-field-enabled nonreciprocal thermal radiation [28], WSM twist-induced thermal radiation [29], three-body Weyl system thermal radiation [30] and so on. In addition, the WSM inherently supports nonreciprocal surface modes and does not require an external magnetic field, which greatly facilitates techniques and preparation [31–33]. Based on the attainment of unidirectional transmission, the process to achieve easier and convenient control of the heat flux transfer direction without particularly complex materials and device preparation processes has become a major problem. However, to date, most modulation methods require the construction of nanometre-sized metamaterial structures to achieve anisotropic patterns, limiting the development and application of current near-field heat flux control schemes.

Due to the above problems, we propose a scheme to achieve the transfer of near-field heat flux by two parallel plates placed at a certain distance. In this scheme, a heterogeneous graphene structure supported by a WSM substrate with a Voigt configuration and a WSM with a twist angle constituted the entire heat transfer system. We found that with a drift current applied to the graphene surface, there was heat flux transfer between two plates when the interplate temperature difference equalled zero. When the distance between the nodes was zero and no temperature difference was present between the two plates, the net heat flux flowed from the graphene heterostructure to the torsional Weyl metal plate. In addition, since the Weyl semimetal inherently supported the nonreciprocal surface modes, the transfer direction of the net heat flux could be regulated by adjusting the size and direction of Weyl node separation. Moreover, the magnitude and direction of the near-field heat flux between the twist Weyl semimetal and the graphene heterostructure could be adjusted by the relative twist angle. Therefore, thermal radiation schemes based on this simple structure are promising for feasible applications in micro/nanothermal radiation devices.

## 2 Theory and method

**Figure 1:**
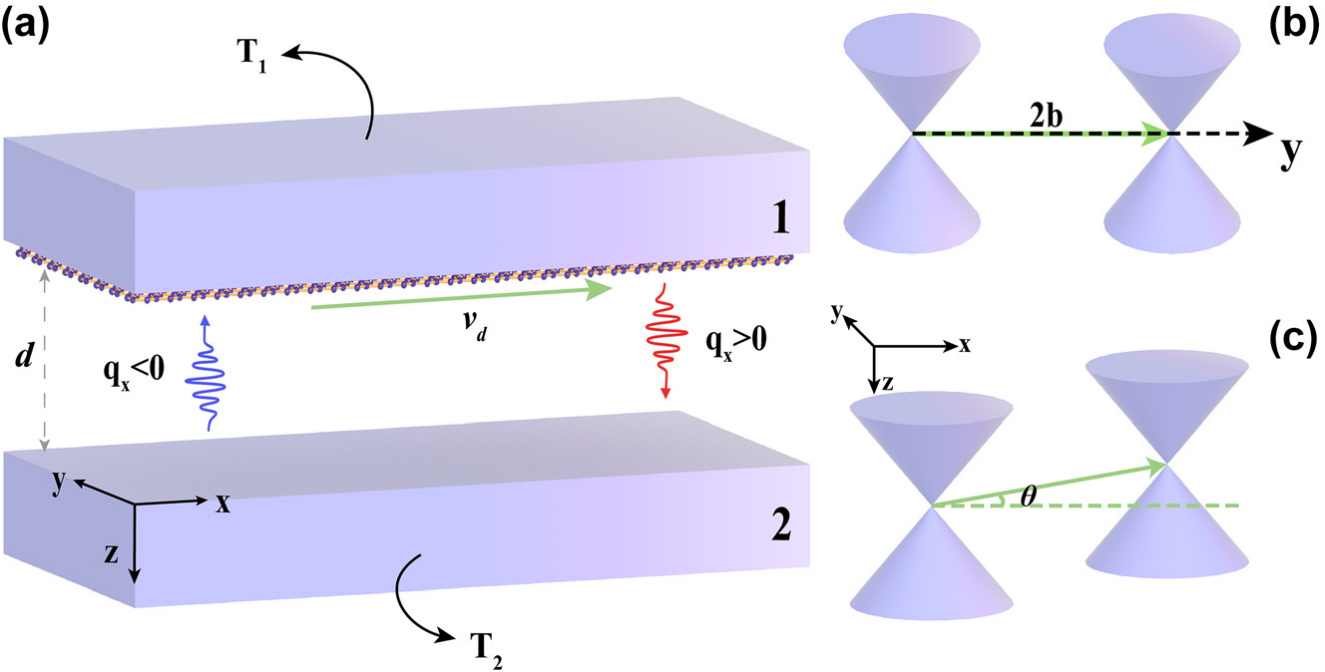
Two-body near-field heat transfer system model. (a) Schematic diagram of a near-field energy transfer system consisting of two parallel plates. (b) Schematic diagram of Weyl node separation in the WSM (i.e., the reciprocal substrate of the upper plate) along the positive direction of the *y*-axis with a twist angle. (c) Schematic diagram of Weyl node separation in the T-WSM of the lower plate in the *x*–*y* plane at a certain twist angle in the positive direction of the *x*-axis.

We consider a simple structure composed of two parallel plates separated by a certain gap, as shown in Figure 1. The upper plate is a graphene heterostructure supported by a WSM substrate and the lower plate is a WSM flat plate with a twist angle. This structure constitutes a system for the near-field energy transfer between the two plates, and the two WSM tablets are semi-infinite. The space between two plates is filled by vacuum, and the distance between them is denoted as *d*. For convenience and clarity, the graphene heterostructure supported by the WSM substrate in Figure 1 is denoted as Object 1; the twist Weyl semimetal (T-WSM) is expressed as Object 2. Accordingly, the temperatures of Object 1 and 2 are written as *T*_1_ and *T*_2_, respectively. For graphene, its thickness is denoted as *d*_*δ*_, and its initial chemical potential is set to 0.1 eV. Furthermore, a current is applied to the graphene at a drift velocity of *v*_*d*_ = 0.3*v*_*F*_, which enables the nonreciprocity of the plasma on the graphene surface to be neglected and causes the near-field energy transfer between the two plates even in the absence of a temperature difference [34]. In the WSM, the Weyl node separation in momentum space causes the anomalous Hall effect. Therefore, that the Weyl node separation in the graphene-covered WSM substrate is assumed to move along the positive direction of the *y*-axis; thus, the nonreciprocity of the WSM can be disregarded. Although the substrate WSM does not exhibit nonreciprocity, the choice of magnetic Weyl semimetals as the substrate is still necessary. This is because the twist angle (*θ*) in our study refers to the angle between the separation wavevectors **2b** of two pairwise Weyl nodes in the upper and lower Weyl semimetal plates. In addition, the presence of magnetic Weyl semimetal plates can also serve as a control for the twist magnetic Weyl semimetal plates. The Weyl node separation of Object 2 occurs in the *x*–*y* plane and forms a twist angle (*θ*) with the positive direction of the *x*-axis. Clearly, the Weyl node separation is along the positive direction of the *x*-axis at *θ* = 0°. Notably, the definition of the twist angle indicates that its existence depends on the pairwise Weyl node separation spacing of the twist magnetic Weyl semimetals. Furthermore, when the separation distance satisfies *b* ≠ 0, the presence of a twist angle can fine-tune the direction of the energy transfer in the near field.

Based on the fluctuational electrodynamics we can express the heat-transfer coefficient (HTC) *H* (i.e., the radiative heat conductance per unit area) between two parallel plates as follows:

(1) $$H=\overset{\infty }{\underset{0}{\int }}\frac{d\omega }{2\pi }\int \frac{d^{2}q}{{\left(2\pi \right)}^{2}}n\left(\omega ,q_{x}\nu_{d}\right)\xi \left(\omega ,q,b,\nu_{d}\right).$$

where $q=\left(q_{x},q_{y}\right)$ is the wave vector in the plane, *ω* is the angular frequency, and *n* is the photon occupation number. By applying a current to the graphene surface at a drift velocity of *ν*_*d*_, a difference in the Bose–Einstein distribution is created between the two plates. Then, *n* can be expressed as follows:

(2) $$\begin{array}{ll} n\left(\omega ,q_{x}\nu_{d}\right) & ={\left[e^{\hbar \left(\omega -q_{x}\nu_{d}\right)/k_{B}T_{1}}-1\right]}^{-1}\\ & \;-{\left[e^{\hbar \omega /k_{B}T_{2}}-1\right]}^{-1}. \end{array}$$

where *T*_1_ and *T*_2_ are the temperatures of Object 1 and 2, respectively, and *k*_*B*_ is the Boltzmann constant. Based on the above equation, in the case of a current drifting on the graphene surface at a velocity of *ν*_*d*_ along the positive direction of the *x*-axis, the nonequilibrium fluctuations generated by this drifting current would cause finite near-field heat flux transfer even if there is no temperature difference between the two plates, i.e., *T*_1_ = *T*_2_. To neglect the effect of *ω* < *q*_*x*_ *ν*_*d*_, we choose a vacuum gap of no less than 10 nm [35–37]. When *q*_*x*_ > 0, *n* is positive, and the energy transfer occurs from the graphene heterostructure to the twist-Weyl metal plate; when *q*_*x*_ < 0, n is negative, and the reverse occurs. Even if a temperature difference exists between the two plates of the two-body system, the near-field energy transfer is not necessarily more drastic than that in the case without a temperature difference. According to Eq. (1) and Eq. (2), if the temperature of the two-body system in the no-temperature-difference case is smaller than the minimum temperature in the temperature-difference case, then the near-field energy transfer in the temperature-difference case is more drastic than that in the no-temperature-difference case.

The photon transport coefficient *ξ* in Eq. (1) can be expressed as follows [38]:

(3) $$\xi \left(\omega ,q,b,\nu_{d}\right)=\left\{\begin{array}{c} \mathrm{Tr}\left[\left(I-R_{2}^{†}R_{2}\right)D\left(I-R_{1}R_{1}^{†}\right)D^{†}\right],\;\;\;\;\;\;\;\;\;\;\;\;\;\;\;\;\;\;\;\;\;\;\;\;q<k_{0}\\ \mathrm{Tr}\left[\left(R_{2}^{†}-R_{2}\right)D\left(R_{1}-R_{1}^{†}\right)D^{†}\right]e^{-2\left|\beta_{0}\right|d},\;\;\;\;\;\;\;\;\;\;\;\;\;\;q>k_{0}\; \end{array}\right.$$

where $D={\left(I-R_{1}R_{2}e^{2i\beta_{0}d}\right)}^{-1}$ and *I* is the unit matrix. The out-of-plane wave vector *β*_0_ in the air is of size $\beta_{0}=\sqrt{k_{0}^{2}-q^{2}}$ , where *k*_0_ = *ω*/*c* and $q=\left|q\right|$ . *R*_*N*_ is a 4 × 4 reflection coefficient matrix of the flat plate *N*. The specific calculation can be found in Ref. [8], where the near-field and far-field regions are distinguished by *q* > *k*_*0*_ and *q* < *k*_*0*_, respectively. According to Eq. (1) and Eq. (2), the heat flux flowing from the graphene heterostructure to the twist-WSM plate is defined as a positive energy flux; otherwise, it is called a negative energy flux. Here, we further introduce an energy transfer function, which can be expressed as follows:

(4) $$Z\left(\omega ,q,b,v_{d}\right)=\hbar \omega n\left(\omega ,q_{x}v_{d}\right)\xi \left(\omega ,q,b,v_{d}\right).$$

This energy transfer function reflects the transport of the near-field heat flux at a given angular frequency *ω* and wave vector ***q***.

## 3 Results and discussion

**Figure 2:**
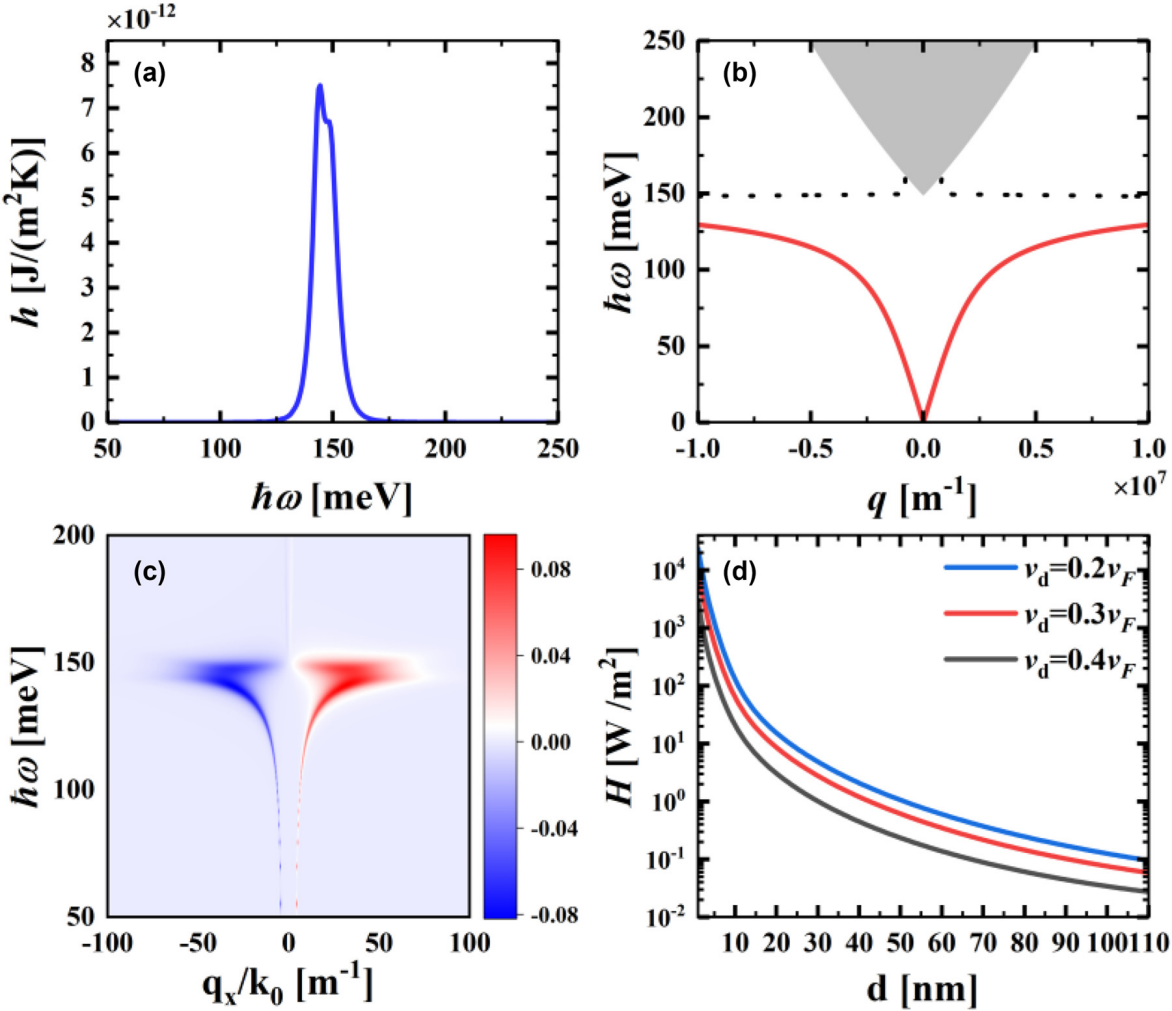
Consideration of only the near-field energy transfer in the case of the graphene nonreciprocal photon occupation number. (a) Spectra of *h* as a function of frequency. (b) Surface dispersion curves of the WSM. The grey area indicates bulk plasmon polaritons, the black dashed line is the asymptote of the surface plasmon polaritons, and the solid red line indicates the dispersion of the surface mode. (c) Energy transmission function Z (in units of meV) against *q*_*x*_/*k*_0_ and *ℏω*. (d) Relationship between energy flux *H* and vacuum gap thickness *d* for different *d* scenarios. Where *b* = 0 m, *d* = 50 nm, *q*_*y*_ = 0, *v*_*d*_ = 0.3*v*_*F*_ and *T*_1_ = *T*_2_ = 300 K.

In this section, we initially discuss the near-field heat flux transfer between two flat plates in the absence of a temperature difference (*T*_1_ = *T*_2_ = 300 K) and Weyl node spacing (*b* = 0 m). When the Weyl nodes in momentum space are not separated, both the WSM material and the transmission coefficient satisfy Lorentz reciprocity. Therefore, we only need to consider the effect of the nonreciprocal photon occupation number caused by the drifting current on the near-field energy transfer. To facilitate the study, we define the spectral function $h\left(\omega \right)$ by $H=\int_{0}^{\infty }h\left(\omega \right)/2\pi d\omega$ . For cases *ω* > *q*_*x*_ν_*d*_ and *q*_*x*_ > 0, the positive wave vector +*q*_*x*_ in graphene plays a dominant role in energy transfer due to the photon occupation number *n*(*ω*,*q*_*x*_ *ν*_*d*_) > n(*ω*,−*q*_*x*_ *ν*_*d*_). As a result, the heat flux flows from the graphene heterostructure to the T-WSM, leading to a positive net heat flux value, as shown in Figure 2(a). The near-field heat flux transfer mechanism is detailed in the dispersion curves of the T-WSM flat plate, as shown in Figure 2(b). We find that the dispersion curves of surface plasmon polaritons and bulk plasmon polaritons at the positive and negative wave vectors are symmetrically distributed, which means that they satisfy Lorentz reciprocity. To observe the heat flux transfer phenomenon in the near-field region more intuitively, we illustrate the energy transfer function in the case of the Weyl node separation distance *b* = 0 in Figure 2(c). The red color in the figure indicates the forward transfer of energy; specifically, the heat flux is transferring from the graphene heterostructure to the T-WSM; the blue color indicates the reverse transfer of energy. The shades of red and blue indicate the intensity of the energy transfer. In the case where only the graphene nonreciprocal photon occupation number is considered, the heat flux transfer is determined by the positive wave vector +*q*_*x*_. Based on these findings, we further present the variation in the near-field heat transfer coefficient as a function of different vacuum gap thicknesses (*d*) at different drift velocities, as shown in Figure 2(d). In the near-field region, the contribution of the evanescent waves results in a significant enhancement of the heat transfer coefficient, which surpasses that of the blackbody radiation by several orders of magnitude. This result also demonstrates the advantage of near-field thermal radiation over blackbody radiation. However, the heat transfer coefficient tends to monotonically decrease with increasing vacuum gap thickness *d* because the contribution of the evanescent waves is highly constrained in the far-field region [39]. Moreover, the decrease in the drift velocity *ν*_*d*_ of the current on graphene positively contributes to the increase of the heat transfer coefficient. This drift velocity provides a way to achieve dynamic manipulation of the heat transfer coefficient.

**Figure 3:**
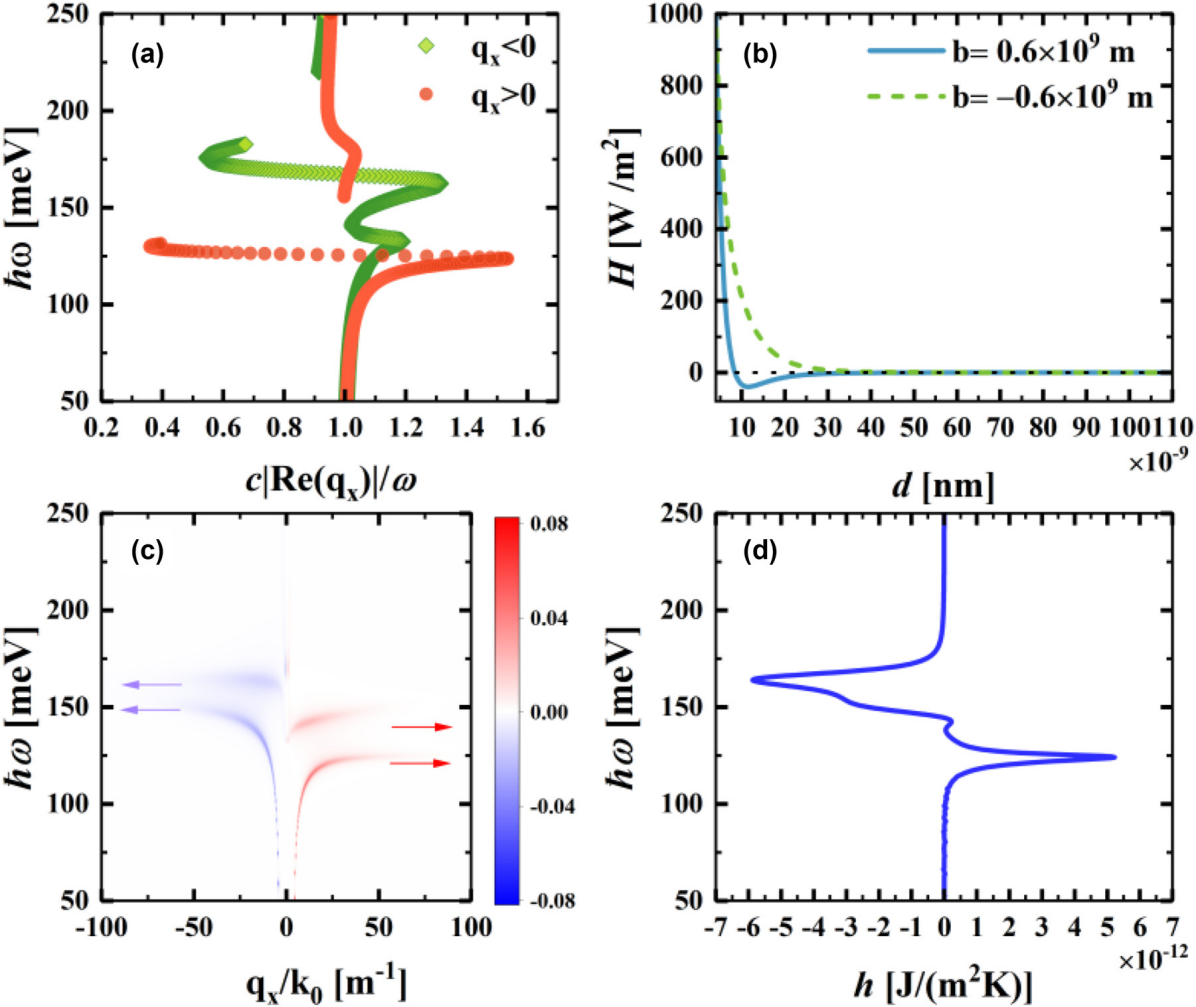
Near-field energy transfer case under the joint action of the nonreciprocal photon occupation number and the nonreciprocal surface modes. (a) Dispersion of the surface modes of the T-WSM in the Faraday configuration at *b* = 0.6 × 10^9^ m. (b) Relationship between energy flux *H* W/m^2^ and vacuum gap thickness *d* at *b* = 0.6 × 10^9^ m and *b* = −0.6 × 10^9^ m. (c) Energy transmission function Z (measured in meV) against *q*_*x*_/*k*_0_ and *ℏω*. Energy *ℏω* shown by the arrow is the same as the corresponding energy of the peak in Figure 3(d). (d) Spectrum of *h* at *b* = 0.6 × 10^9^ m and *d* = 50 nm. The parameters have the same values as those in Figure 2.

Since the effect of the nonreciprocal photon occupation number of graphene on the energy transfer is understood, we further discuss the near-field heat flow transfer in the absence of a temperature difference, under the joint action of the nonreciprocal photon occupation number due to drift currents and nonreciprocal surface modes supported by the T-WSM. The separation of WSM nodes lead to large anomalous Hall effects, which induce nonreciprocal surface patterns that break the constraints of the reciprocal system. The nonreciprocity is mainly occurs because the damping intensity in the −*q*_*x*_ wave vector region is larger than that in the +*q*_*x*_ wave vector region. We consider the disconnected region as a region of bulk plasmon polaritons, as shown in Figure 3(a). Since the photon transport coefficients tend to respond differently to positive and negative wave vectors under the influence of the nonreciprocal surface modes supported by the WSM, the node separation of the WSM in momentum space can have a significant effect on the near-field heat transfer. To visualize the near-field heat flux transmission, the variation of the heat transfer coefficient (HTC) with respect to the vacuum gap thickness *d* for the Weyl nodes separation spacing *b* = 0.6 × 10^9^ m and *b* = −0.6 × 10^9^ m are plotted (Figure 3(b)). Although the weakening of the photon tunneling effect in the far-field region leads to an overall decrease in the near-field heat flux transport, the decreasing trend of the heat transfer coefficient varies according to the separation spacing of Weyl nodes. When *b* = −0.6 × 10^9^ m, the heat transfer coefficient tends to monotonically decrease with increasing vacuum gap thickness *d*. The stretching of the WSM at +*q*_*x*_ leads to a synergistic effect between the nonreciprocal photon occupation number and the non-reciprocal surface modes, resulting in heat flux transfer from the graphene heterostructure to the WSM. However, in the case of *b* = 0.6 × 10^9^ m, the variation curve of the heat transfer coefficient initially declines and then increases. When *d* increases from a small value, the heat transfer coefficient shows a rapid decrease from a high level near 1000 W/m^2^. When *d* ≈ 8 nm, the heat transfer coefficient begins to show negative values. To better understand this phenomenon, we plot the energy transfer function $Z\left(\omega ,q,b,\nu_{d}\right)$ and the energy flux spectrum $h\left(\omega \right)$ for the cases of *b* = 0.6 × 10^9^ m and *d* > 8 nm at a certain place (i.e., *d* = 50 nm) are plotted, as shown in Figure 3(c) and (d), respectively. In Figure 3(c), blue and red arrows are used to indicate the peaks of reverse and forward energy transfer, respectively. These arrows correspond exactly to the peaks in Figure 3(d). Asymmetry in the distribution of red and blue colors is easily observed in this figure. This is a result of the varied response of photon transfer coefficients to positive and negative wave vectors under the influence of T-WSM support for nonreciprocal surface modes. Furthermore, Figure 3(d) demonstrates that the negative spectrum of $h\left(\omega \right)$ is not only larger in amplitude than the positive spectrum of $h\left(\omega \right)$ but also has a wider regional range than the latter; this result is consistent with the data shown in Figure 3(c). Therefore, the near-field heat flux transfer coefficient is negative in this case, i.e., the energy is transferring from the graphene heterostructure to the T-WSM. In addition, the nonreciprocal photon occupation number increases with the wave vector *q*_*x*_ in the case of *b* = 0.6 × 10^9^ m and *d* ≲ 8 nm, allowing energy to travel from the graphene heterostructure to the T-WSM. However, when *b* = 0.6 × 10^9^ m and *d* ≳ 8 nm, there would be more polariton damping at −*q*_*x*_ than at +*q*_*x*_ in nonreciprocal surface mode, resulting in a wider energy flux spectrum $h\left(\omega \right)$ at −*q*_*x*_ than in the region of +*q*_*x*_ and thus a reverse transfer of energy from the T-WSM plate to the graphene heterostructure.

**Figure 4:**
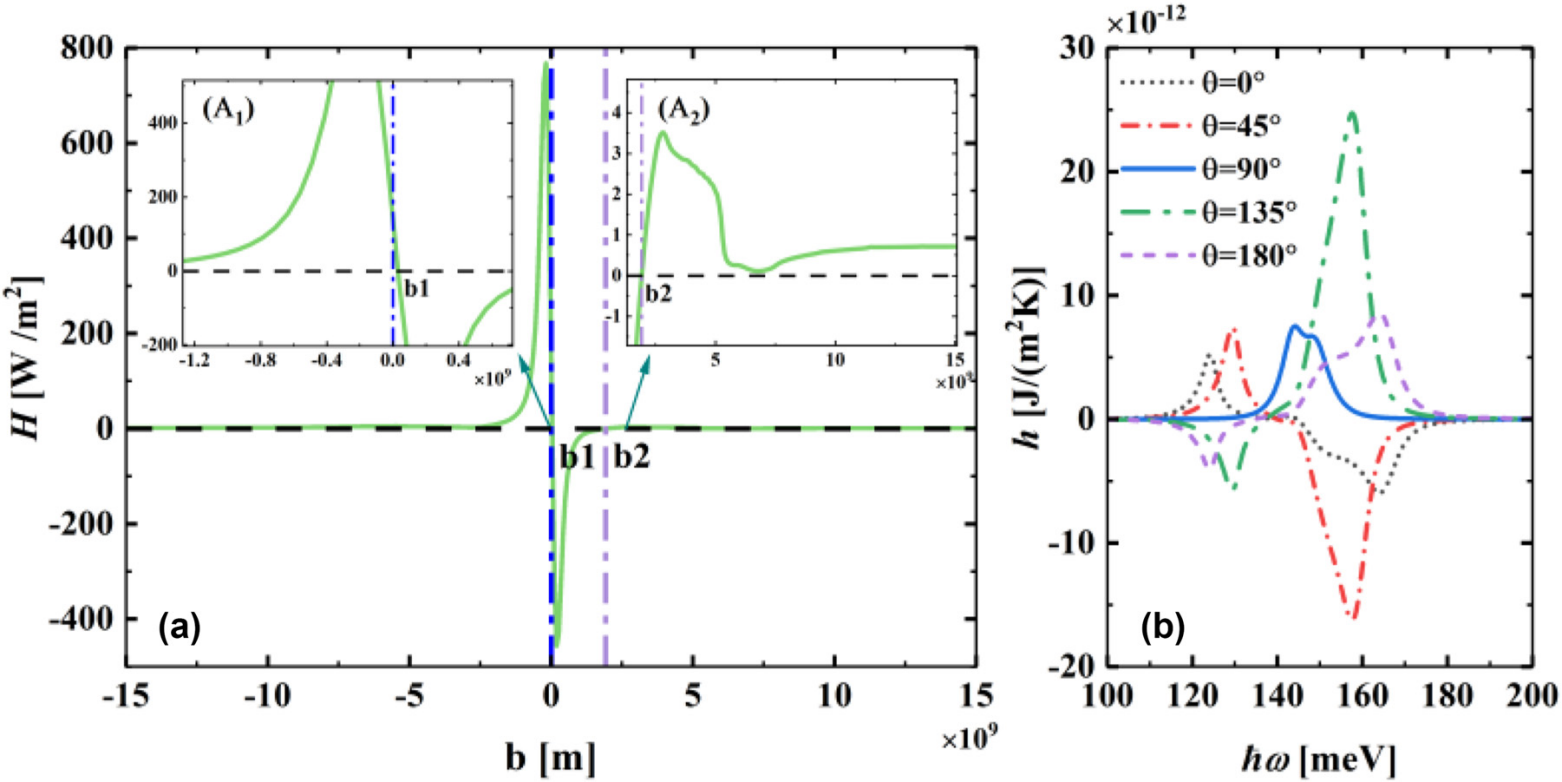
Effect of the heat transfer coefficient with respect to Weyl node spacing and the spectra of *h*. (a) Variation graph of the heat transfer coefficient *H* W/m^2^ as a function of Weyl node separation spacing *b*. (b) Spectrum of *h* as a function of frequency in the presence of different twist angles in the T-WSM. The parameters have the same values as those in Figure 2.

The above discussion shows that the interaction between the nonreciprocal surface modes and the nonreciprocal photon occupation number can determine the direction of the net heat flux transfer. The theory in the second part shows a very close relationship between the Weyl node separation spacing and the twist angle in the WSM materials on the transmission coefficient of this two-body system. Therefore, the study of the effects of the Weyl node separation spacing and the twist angle on the transmission coefficient has great practical significance and can also provide important references for the design of related thermal radiation devices. On this basis, we first discuss the effect of different Weyl node spacings on the transmission coefficient, as shown in Figure 4(a). We focus on the evolutionary pattern around the Weyl node spacing *b* = 0. As the Weyl node separation spacing in momentum space increases from 0 to *b*_1_, the transmission coefficients remain immediately above the *x*-axis and show a monotonic decreasing trend. This occurs because the nonreciprocity of the T-WSM metal is weak at the moment when b starts to increase from 0; additionally, the nonreciprocal photon occupation number caused by the drift current on graphene still has a dominant position in the energy transfer contribution. Consequently, the energy is transferred from the graphene heterostructure to the T-WSM. However, a further increase in the Weyl node separation spacing *b* reverses the transfer direction of the heat flux. As shown in Figure 4(a), a prominent negative heat flow is present in the region of *b*_1_ < *b* < *b*_2_. The mechanism here is similar to that in Figure 3(b). Next, as *b* continues to increase, the near-field transmission coefficient curve increases back to the position above the *x*-axis. There are two main reasons for this phenomenon. On the one hand, in a given frequency range the nonreciprocal nature of the T-WSM diminishes with increasing Weyl node spacing, thus enabling the nonreciprocal photon occupation number of graphene to regain its dominant position in energy transfer. On the other hand, the contribution of the negative wave vector region to energy transfer is suppressed by the strong damping of the surface waves at *q*_*x*_ < 0. The combined effect of these reasons results in the transfer of energy from the graphene heterostructure to the T-WSM. For the near-field region in the range of *b* < 0, the value of the heat transfer coefficient always lies above the *x*-axis and shows a trend of initially increasing and then decreasing with increasing *b*. This phenomenon is the result of the interaction between the nonreciprocal photon occupation number and the nonreciprocal surface modes. In the case of *b* < 0, the stretching of T-WSM surface polaritons occurs at +*q*_*x*_. At this time, the nonreciprocal surface mode of the T-WSM has a positive contribution to the near-field energy transfer, synergizing with the nonreciprocal photon occupation number of graphene to promote the positive heat flux transfer. When the negative separation distance *b* of Weyl nodes is too large, the polaritons on the T-WSM surface become highly irreversible, and the surface polarons in the negative wavevector region are strongly damped, thus inhibiting the near-field energy transfer in the negative wavevector region. In addition, this phenomenon becomes more prominent as the nonreciprocity becomes weak in a given frequency range. For these two reasons, the number of nonreciprocal photon occupancies in graphene regains its dominant position in energy flux transfer, thus allowing the energy to flow from the graphene heterostructure to the WSM. Moreover, we also apply the spectral function $h\left(\omega \right)$ to demonstrate the effect of the twist angle between the wave vector 2*b* and the *x*-axis on the near-field heat flux transmission, as shown in Figure 4(b). When *θ* = 90°, the node separation of the T-WSM in momentum space exhibits reciprocity along the *y*-axis perpendicular to the direction of the drift current. Therefore, the nonreciprocal photon occupation number in graphene is dominant in the energy transfer. This result is similar to the case in Figure 2(a), where the Weyl nodes are not separated. As the twist angle increases from *θ* = 0° to *θ* = 180°, the transport direction of the near-field heat flux undergoes an inverse transition. Notably, the change in the nearfield radiative heat flux caused by the twist angle is not symmetric because the nonreciprocal photon occupation number of graphene has a higher contribution to heat flux transfer in the region of positive wave vectors than in the region of negative wave vectors. As a result, the overall near-field heat flux transfer is dominated by the positive heat flux.

**Figure 5:**
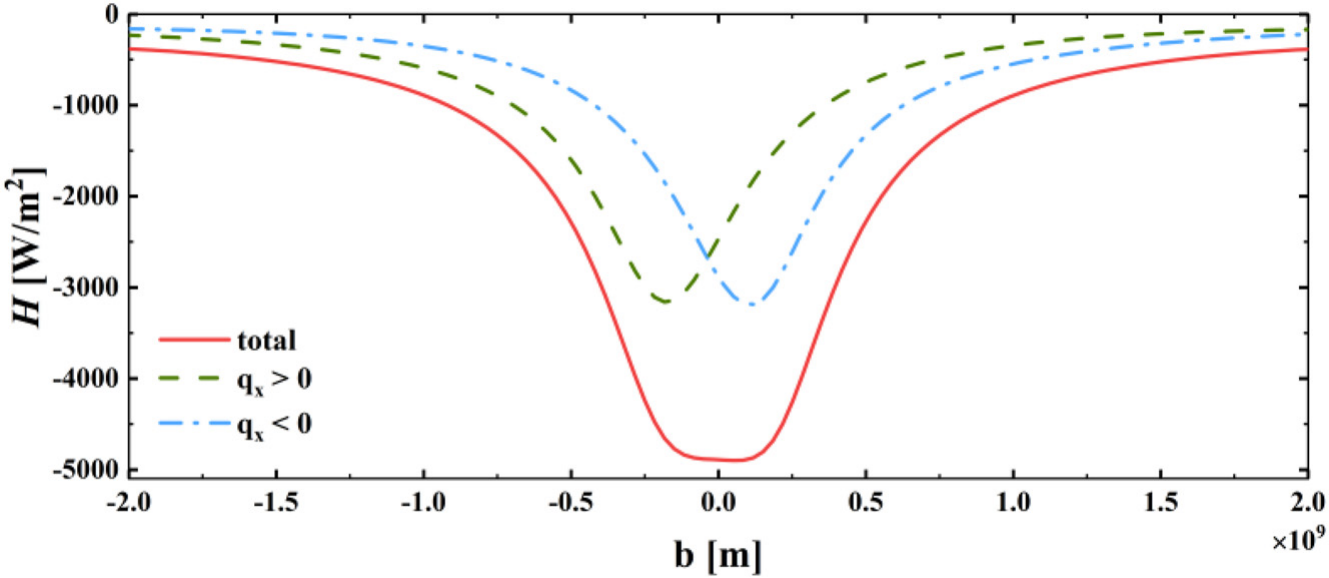
Variation of the heat transfer coefficient *H* as a function of Weyl node separation distance *b* for different *q*_*x*_ scenarios, where *T*_1_ = 300 K, *T*_2_ = 300 K and *d* = 50 nm.

Finally, we discuss the case of thermal radiation without the application of drift current on the graphene surface and with a temperature difference between the upper and lower plates. The other parameters remain the same as the initial parameters in the second part. At this point, we set the temperature of Object 1 and 2 to be *T*_1_ = 300 K and *T*_2_ = 330 K. In this case, the photon occupation number can be expressed as $n\left(\omega \right)={\left[e^{h\omega /k_{B}T_{1}}-1\right]}^{-1}-{\left[e^{h\omega /k_{B}T_{2}}-1\right]}^{-1}$ . On this basis, the effect of the Weyl node separations on the heat transfer coefficient under positive and negative wave vectors is shown in Figure 5. When the Weyl node separations of T-WSM are along the positive direction of the *x*-axis, the heat-transfer coefficient is significantly different in the case of positive wave vector +*q*_*x*_ and negative wave vectors −*q*_*x*_. This results in a difference in the number of electron occupancies in graphene in the positive wave vector region +*q*_*x*_ and negative wave vector region −*q*_*x*_. Furthermore, when the impurity scattering in graphene is weak, the ultrahigh electron mobility of graphene causes this occupancy number difference to generate induced currents along the *x*-axis. A necessary condition for this thermoelectric effect is the presence of at least one nonreciprocal flat plate, similar to the conversion of thermal energy to mechanical energy in Casimir heat engines [40, 41]. The pseudo-vector 2*b* in momentum space can regulate the magnitude and direction of the induced electric current, thus achieving the near-field thermoelectric effect. In addition, the nonreciprocity of the T-WSM would disappear at *b* = 0, making it impossible to generate induction currents.

## 4 Conclusions

In conclusion, we investigated the current-induced tunable near-field energy transfer between the twist magnetic Weyl semimetals and graphene. In the absence of temperature difference, the magnetic Weyl semimetals showed reciprocity when no nodal separation in the twist-magnetic Weyl semimetal was present. At this moment, the drift current on the graphene surface determined the magnitude and direction of the near-field energy transfer. However, when the nodes in the twist-magnetic Weyl semimetal became separated, the magnitude and direction of the near-field energy transfer were jointly determined by the nonreciprocal surface modes supported by the twist-magnetic Weyl semimetal and the nonreciprocal photon occupation number in graphene, and the presence of the twist angle in the magnetic Weyl semimetal could also modulate the direction of near-field energy transfer. Furthermore, we also discussed and analysed the interesting thermoelectric effects based on the nonreciprocal surface modes to generate induced currents. Our study provides an achievable pathway for unidirectional thermal radiation and nanoscale thermal management.

## Supplementary Material

Supplementary Material Details

Supplementary Material Details
