# Supplementary material for: Drift current-induced tunable near-field energy transfer between twist magnetic Weyl semimetals and graphene
Source: Nanophotonics. 2023 Oct 5;12(20):3911–20. doi: 10.1515/nanoph-2023-0345 (PMC11502063; doi:10.1515/nanoph-2023-0345)
Supplement: Supplementary file 1 — Supplementary Material Details [file j_nanoph-2023-0345_suppl_001.docx]

Supplementary information

**Drift Current-Induced Tuneable Near-Field Energy Transfer Between Twist Magnetic Weyl Semimetals and Graphene**

Qijun Ma^1^, Xue Chen^1^, Qisen Xiong^1^, Leyong Jiang^1,*^, Yuanjiang Xiang^2,**^

*^1^School of Physics and Electronics, Hunan Normal University, Changsha 410081, China;*

*^2^School of Physics and Electronics, Hunan University, Changsha 410082, China*

^*^Email: jiangly28@hunnu.edu.cn

^**^Email: xiangyuanjiang@126.com

**Supplementary 1: Theoretical derivations of the graphene conductivity**


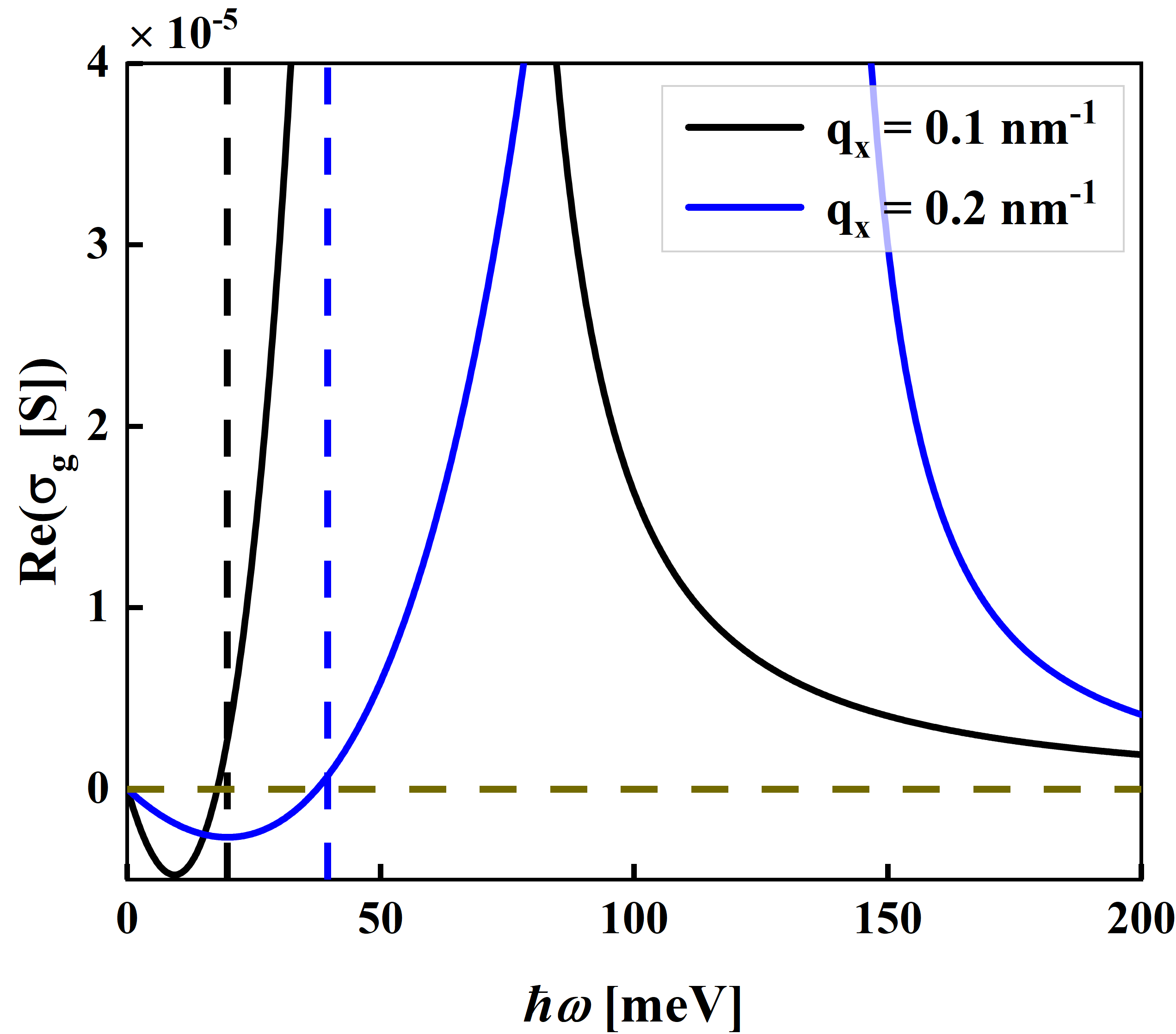


Fig. S1. Real part of the graphene conductivity varies with for different $q=\left(q_{x},0\right)$ scenarios with currents drifting along the x-axis. Here, $\mu_{g}=0.1\text{ eV}$, $\gamma_{g}=3.7\text{ meV}$, $\nu_{d}=0.3\nu_{F}$ and $T=300K$. The vertical dashed part represents $\omega=q_{x}\nu_{d}$ corresponding to different $q_{x}$ values.

In the near-field heat transfer model, for a graphene plate with currents drifting on its surface along the x-axis, the polarization function related to angular frequency $\omega$ and planar wave vector $q=\left(q_{x},q_{y}\right)$ is expressed as follows [1, 2]:

(S1)

where $\mu(T)=2k_{B}T\ln[2\cosh(\frac{\mu_{g}}{2k_{B}T})],$ T is the temperature, $\mu_{g}$ is the chemical potential, $\gamma_{g}$ is the damping parameter, $\nu_{d}$ is the drift current, and $\nu_{F}$ is the Fermi velocity. Then, the bulk conductivity of graphene will is expressed as follows:

$\sigma_{g}(\omega,q_{x},q_{y})=\frac{ie^{2}\omega}{q^{2}}\Pi(\omega,q_{x},q_{y})$ (S2)

where $q_{x}$ and $q_{y}$ are the planar wave vectors along the x and y directions, respectively, and $q^{2}=q_{x}^{2}+q_{y}^{2}$. The graphene plate is considered an ultrathin monolayer film with a finite thickness ($d_{\delta}=0.3 nm$). Therefore, the dielectric constant of this uncovered monolayer graphene can be expressed as follows [3, 4]:

$\varepsilon_{1}=1+\frac{i\sigma_{g}}{\varepsilon_{0}\omega\Delta}.$ (S3)

Based on Equation (S2), Fig. S1 shows the variation curve of the real part of graphene’s conductivity with under different $q=\left(q_{x},0\right)$ conditions. The real part of graphene’s conductivity becomes negative at frequencies below $q_{x}v_{d}$. This negative conductivity region can produce optical gains and negative Landau damping [1, 5].

**Supplementary 1: Theoretical calculation of the magnetic Weyl semimetals**


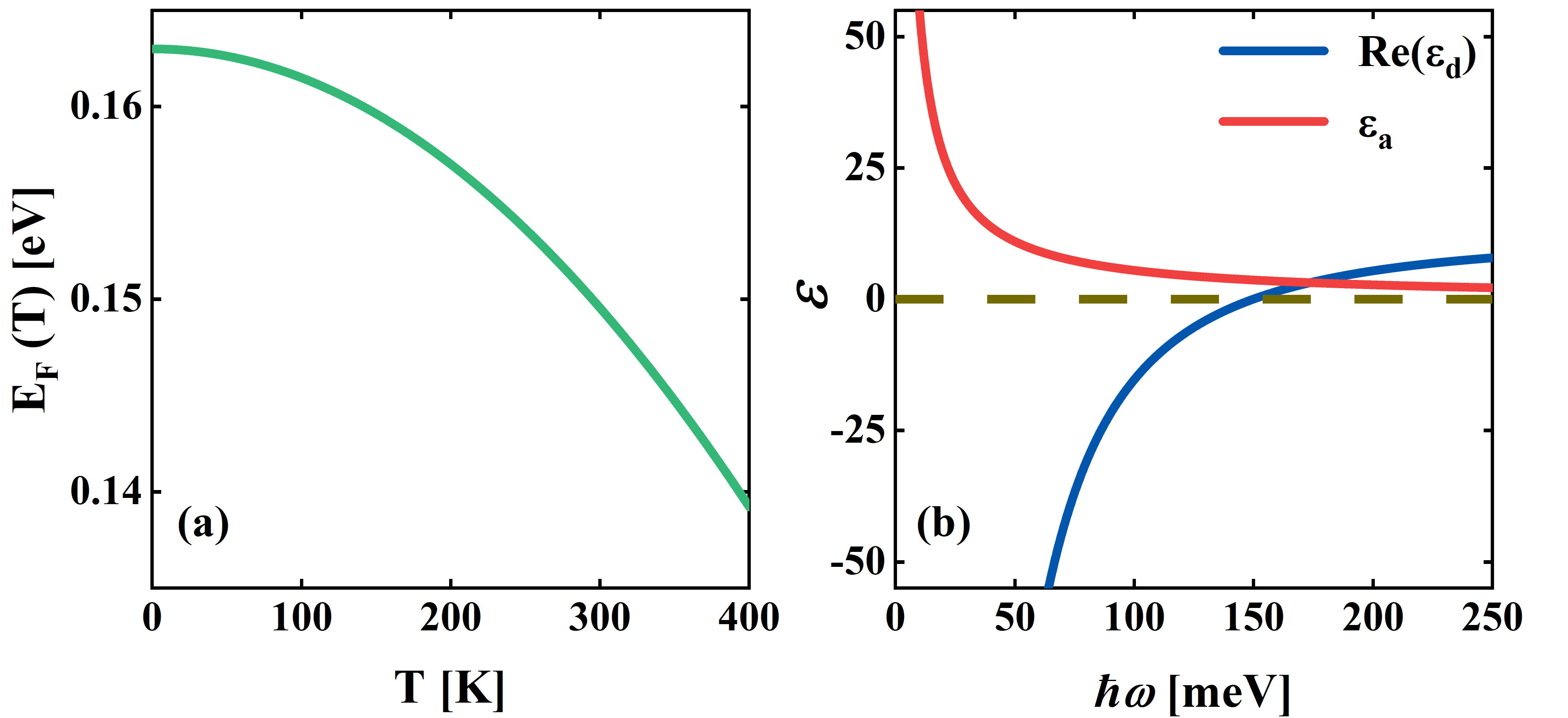


Fig. S2. (a) Variation curve of the chemical potential $E_{\text{F}}(T)\text{ }eV$ with temperature T K in the WSM, where $E_{\text{F}}(0)=0.163\text{ }eV$, $E_{F}(300\text{K})=0.150\text{ }eV$ and $E_{F}(330\text{K})=0.147\text{ }eV$ (b) Variation curves of the real parts of the dielectric tensor’s nondiagonal and diagonal terms in the WSM with respect to .

For a WSM that breaks the time-reversal symmetry, the presence of Weyl nodes changes the electromagnetic effect. Therefore, the electric displacement field of the WSM in the frequency domain can be expressed as follows:

(S4)

where $\varepsilon_{0}$ is the vacuum dielectric constant and $\varepsilon_{d}=\varepsilon_{b}+i\sigma/\omega$ is the dielectric constant of the main diagonal of the Weyl semimetals. Among them, $\varepsilon_{b}$ is the dielectric constant of the background material and $\sigma$ is the bulk conductivity of the WSM. From Eq. (S4), $b_{0}$ produces a chiral magnetic effect and $b$ generates an anomalous Hall effect, thus resulting in a nondiagonal term in the dielectric tensor and enabling the existence of nondiagonal surface modes in the WSM. Since this nonreciprocity does not require an external magnetic field, it is entirely different from the cyclotron mechanism in magneto-optical materials. We consider that wave vector $2b$ in the momentum space spreads along the x-axis direction ($b=b\hat{q}_{x}$) and that there is a time-reversal symmetric system of $b_{0}=0$. Through simplification, we have $D\text{=}\varepsilon_{0}\bar{\bar{\varepsilon}}E$ in Cartesian coordinates. Then, the dielectric tensor $\bar{\bar{\varepsilon}}\left(\omega\right)$ is represented as follows:

$\bar{\bar{\varepsilon}}\left(\omega\right)=\left[\begin{matrix} \varepsilon_{d} & 0 & i\varepsilon_{a} \\ 0 & \varepsilon_{d} & 0 \\ -i\varepsilon_{a} & 0 & \varepsilon_{d} \end{matrix}\right],$ (S5)

with ; notably $\varepsilon_{a}$ can approach the size of $\varepsilon_{d}$ in the infrared region, which is very interesting in thermal applications. For WSM, the implicit dispersion relation of surface modes to wave vector $q_{x}$ is as follows:

$\varepsilon_{eff}\sqrt{\left(\omega/c\right)^{2}-q_{x}^{2}}+\sqrt{\varepsilon_{eff}\left(\omega/c\right)^{2}-q_{x}^{2}}+i\varepsilon_{a}q_{x}/\varepsilon_{d}=0.$ (S6)

where $\varepsilon_{eff}=\varepsilon_{d}-\varepsilon_{a}^{2}q_{x}/\varepsilon_{d}$. According to Eq. (S6), the presence of $\varepsilon_{a}$ breaks the Lorentz reciprocity and thus allows the WSM to support nonreciprocal surface modes.

We obtain the bulk conductivity $\sigma$ by applying the Kubo-Greenwood formalism in the stochastic phase approximation in a two-band model with spin simplification, which, after simplification, is expressed as follows:

(S7)

where is the effective fine-structure constant with the Fermi velocity $\nu_{\text{F}}$, $\text{Ω}=\omega+i\tau^{-1}$ is the plural frequency, $\tau^{-1}$ is the scattering rate corresponding to the Drude damping, $G(E)=n(-E)-n(E)$ with the Fermi distribution function $n(E)$, $g$ is the number of Weyl nodes, $E_{\text{F}}(T)$ is the chemical potential that varies with temperature T and $E_{c}$ is the cutoff energy. Therefore, the diagonal term can be expressed as follows: $\varepsilon_{\text{d}}=\varepsilon_{\text{b}}+i\frac{\sigma}{\omega}$，where $\varepsilon_{\text{b}}$ is the background permittivity accounting for the contributions from all bands below the Dirac cone. According to Refs. [6, 7], the initial parameters in the main text are set as $\varepsilon_{b}=6.2$, $b=0.6\times10^{9}\text{ m}$, $g=2$, $\nu_{\text{F}}=0.83\times10^{5}\text{ m/s}$, $\tau=1000\text{ fs}$ and $E_{\text{F}}=0.15\text{ eV}$. When the temperature satisfies $T=300 K$, $E_{\text{c}}=3E_{\text{F}}$. In Fig. S2(b), $\varepsilon_{\text{d}}$ and $\varepsilon_{a}$ are the real parts of the diagonal elements in the dielectric tensor.

When the direction of the Weyl node separation in the magnetic Weyl semimetal deviates from the x-axis by a certain twist angle, the dielectric tensor becomes the following:

(S8)

where is the transpose symbol and is the Eulerian rotation matrix and defined as follows:

(S9)

The implicit dispersion relation for the twisted magnetic Weyl semimetal is given by the following equation

$\varepsilon_{eff}\sqrt{\left(\omega/c\right)^{2}-q_{x}^{2}}+\sqrt{\varepsilon_{eff}\left(\omega/c\right)^{2}-q_{x}^{2}}+i\cos\theta\varepsilon_{a}q_{x}/\varepsilon_{d}=0.$ (S10)

According to Equation (S10), clearly, the dispersion becomes highly irreversible due to the existence of off-diagonal elements $\varepsilon_{a}$, such that the twisted magnetic Weyl semimetal breaks the Lorentz reciprocity. When we set the twist angle to $\theta\text{=9}0^{\circ}$ for the magnetic Weyl semimetal in the substrate of the graphene heterostructure, the influence of the off-diagonal term in the dispersion relation is eliminated; thus, we can disregard the nonreciprocity in the substrate WSM. In addition, the chemical potential of the Weyl semimetal is closely related to temperature and is caused by linear dispersion due to its small and nonconstant density of states. We can calculate the temperature-related chemical potential through charge conservation by the following equation [8]:

$E_{F}(T)=\frac{2^{1\text{/}3}[9E_{F}(0)^{3}+\sqrt{81E_{F}(0)^{6}+12\pi^{6}k_{B}^{6}T^{6}}]^{2\text{/}3}-2\pi^{2}3^{1/3}k_{B}^{2}T^{2}}{6^{2\text{/}3}[9E_{F}(0)^{3}+\sqrt{81E_{E}(0)^{6}+12\pi^{6}k_{B}^{6}T^{6}}]^{1/3}}.$ (S11)

where, $E_{\text{F}}(0)=0.163\text{ eV}$. Then, $E_{F}(300\text{K})=0.150\text{ eV}$ and $E_{F}(330\text{K})=0.147\text{ eV}$. To observe the effect of temperature on the chemical potential, the change in the chemical potential with respect to temperature is plotted, as shown in Fig. S2 (a).

**References**

1. J. Peng and J.-S. Wang, “Current-Induced Heat Transfer in Double-Layer Graphene,” arXiv:1805.09493, 2019.
2. G. Tang, L. Zhang, Y. Zhang, J. Chen, and C. T. Chan, “Near-Field Energy Transfer between Graphene and Magneto-Optic Media,” *Phys. Rev. Lett*., vol. 127, p. 247401, 2021.
3. A. Vakil and N. Engheta, “Transformation Optics Using Graphene,” Science, vol. 332, pp. 1291–1294, 2011.
4. M. Lim, S. S. Lee, and B. J. Lee, “Near-field thermal radiation between graphene-covered doped silicon plates,” *Opt. Express*, vol. 21, p. 22173, 2013.
5. T. A. Morgado and M. G. Silveirinha, “Negative Landau Damping in Bilayer Graphene,” *Phys. Rev. Lett*., vol. 119, p. 133901, 2017.
6. B. Zhao, C. Guo, C. A. C. Garcia, P. Narang, and S. Fan, “Axion-Field-Enabled Nonreciprocal Thermal Radiation in Weyl Semimetals,” *Nano Lett*., vol. 20, pp. 1923–1927, 2020.
7. G. Tang, J. Chen, and L. Zhang, “Twist-induced control of near-field heat radiation between magnetic Weyl semimetals,” *ACS Photon*., vol. 8, pp. 443–448, 2021.
8. P. E. C. Ashby and J. P. Carbotte, “Chiral anomaly and optical absorption in Weyl semimetals,” *Phys. Rev. B*, vol. 89, p. 245121, 2014.
